# Modified fourth-generation minimally invasive technique with a modified K-wire fixation technique for hallux valgus: a biomechanical and clinical study

**DOI:** 10.1186/s12891-026-10151-1

**Published:** 2026-07-28

**Authors:** Pu Chen, Hua Guan, Enhui Feng, Jiachang Liang, Xinying He, Weiming Huang, Yiyin Xu, Jiewei Xie

**Affiliations:** 1 Department of Trauma and Foot-Ankle Surgery, Guangdong Provincial Hospital of Chinese Medicine, Guangzhou, 510120 China; 2 The Second Clinical College of Guangzhou University of Chinese Medicine, Guangzhou University of Chinese Medicine, Guangzhou, China

**Keywords:** Hallux valgus, Minimally invasive surgery, Four-dimensional correction, K-wire, Modified technique, Finite element analysis

## Abstract

**Background:**

Minimally invasive techniques have become prominent in hallux valgus (HV) correction. However, prevalent third and fourth-generation methods using the “in-out-in” screw fixation often involve extensive intraoperative fluoroscopy, a steep learning curve, and higher costs. This study introduces and evaluates a modified fourth-generation technique featuring an extra-articular transverse osteotomy combined with a modified Kirschner wire (K-wire) fixation technique and the concept of “fourth-dimensional correction” (dynamic and static assessment).

**Methods:**

The study comprised two parts. First, a finite element analysis (FEA) model was developed to compare the biomechanical performance (bone/implant stress, stability) of the modified K-wire fixation technique versus the standard “in-out-in” K-wire fixation at different lateral translation distances (4, 8, 12 mm). Second, a prospective case series of 108 patients (164 feet) with mild to severe HV who underwent the modified technique was analyzed. Outcomes included radiographic parameters (HVA, IMA, DMAA), the AOFAS score, complications, and intraoperative fluoroscopy usage.

**Results:**

FEA demonstrated that the modified K-wire fixation technique provided stability comparable to the “in-out-in” technique while exhibiting significantly lower stress on the K-wires, suggesting a reduced risk of pin fracture. Clinically, all osteotomy sites achieved bony union. Significant improvements were observed in the entire cohort: mean HVA corrected from 36.93°±9.32° to 9.57°±4.88°, mean IMA from 14.86°±3.82° to 6.08°±3.43°, and mean AOFAS score from 61.26 ± 6.97 to 88.90 ± 4.68 (all *P* < 0.01). Excellent correction was maintained across all severity subgroups. The mean intraoperative fluoroscopy shots were 4.14 ± 3.04 per foot, showing a distinct learning curve that stabilized after approximately 25 cases, with usage significantly lower than rates reported for traditional “in-out-in” methods.

**Conclusion:**

The modified fourth-generation minimally invasive technique with the modified K-wire fixation technique is biomechanically sound and clinically effective. It provides reliable radiographic and functional outcomes for HV of varying severities while substantially reducing intraoperative radiation exposure, offering a valuable alternative in the minimally invasive HV correction arsenal.

**Supplementary Information:**

The online version contains supplementary material available at https://doi.org/10.1186/s12891-026-10151-1.

## Introduction

Minimally invasive hallux valgus (HV) correction surgery has gradually gained favor among foot and ankle surgeons due to its advantages of less pain, quick recovery, minimal trauma, no damage to the metatarsophalangeal (MTP) joint, and satisfactory correction of HV deformity [1–4]. The third-generation minimally invasive HV correction technique has developed from the Chevron osteotomy, which is performed percutaneously using 3 to 4 small incisions under fluoroscopy to complete the osteotomy and fix the osteotomy ends with screws [5]. Compared to the third-generation technique, the fourth-generation technique places greater emphasis on extra-articular transverse osteotomy of the metatarsal, which has advantages in correcting rotational deformities [6, 7]. However, both the third and fourth-generation techniques do not focus on evaluating the inversion and eversion balance of the first MTP joint in dynamic and static conditions, leading to recurrence of deformity or the development of hallux varus postoperatively. Additionally, both third- and fourth-generation techniques utilize “in-out-in” fixation, a procedure involving a guide pin passed through the medial and lateral cortices of the proximal metatarsal to the metatarsal head, followed by cannulated screw placement. This method requires extensive fluoroscopy, has a steep learning curve, and carries disadvantages including skin irritation, need for secondary removal surgery, and high costs [8, 9]. 

Therefore, we propose a modified fourth-generation technique for HV correction incorporating an extra-articular transverse osteotomy and a modified K-wire fixation technique, where the internal implant passes through the medial cortex of the proximal metatarsal, then through the medial cortex of the distal metatarsal, and finally reaches the lateral cortex of the distal metatarsal—allowing direct visualization and potentially reducing fluoroscopy. This study aims to: (1) compare the biomechanical performance of the modified K-wire fixation versus standard “in-out-in” fixation using finite element analysis; (2) evaluate short-term clinical outcomes and fluoroscopy frequency across different HV grades; and (3) introduce “fourth-dimensional correction”—assessing post-correction hallux position not only statically but also under simulated weight-bearing to identify residual instability requiring adjunctive procedures, thereby minimizing recurrence risk.

## Methods

### Finite element analysis of “In-Out-In” versus modified K-wire fixation

A finite element model was developed based on CT data from a patient with moderate HV (HVA 37°, IMA 15°), simulating a transverse osteotomy with distal fragment lateral translation of 4, 8, and 12 mm [10]. For each translation distance, two fixation configurations were compared: the conventional “in-out-in” technique and the modified K-wire fixation technique. Under a simulated 250 N weight-bearing load, the following parameters were evaluated: stress distribution at the osteotomy site and on the K-wires, three-dimensional displacement of the osteotomy site, change in IMA, and plantar stress distribution. Detailed modeling parameters are provided in the Supplementary Materials.

### Clinical efficacy evaluation: patient data

Collected from March 2023 to February 2024, 108 patients with HV treated with modified fourth-generation minimally invasive techniques combined with K-wires, totaling 164 feet, with an average age of 44.40 ± 14.85 (16–85 years), including 12 males and 96 females, with 52 single feet and 56 double feet. Inclusion Criteria: (1) Primary HV correction using the modified fourth-generation technique with K-wire fixation; (2) failure of conservative treatment; (3) all surgeries performed by the same surgeon. Exclusion Criteria: (1) Previous ipsilateral hallux or first metatarsal surgery; (2) active foot infection; (3) severe peripheral vascular/neurological disorders or poorly controlled diabetes compromising outcomes; (4) inability to comply with follow-up due to neuropsychological impairment. The detailed baseline data is shown in Table 1.

**Table 1:** The table of patient baseline data

	Pre-operation	post-operation
Age (Year)	44.40 ± 14.85(16–85)	/
Sex	Male: 12 Female: 96	/
HVA	36.93 ± 9.32 <30: 38 feet (25.48 ± 3.22) 30–40: 70 feet (34.82 ± 2.88) >40: 56 feet (47.33 ± 5.85)	9.57 ± 4.88* 6.12 ± 2.34* 9.03 ± 4.40* 12.59 ± 4.92*
IMA	14.86 ± 3.82 <13: 53 feet (10.83 ± 1.59) 13–16: 51 feet (14.46 ± 0.92) >16: 60 feet (18.93 ± 2.11)	6.08 ± 3.43* 4.07 ± 2.16* 5.16 ± 2.19* 8.62 ± 3.63*
DMAA	10.77 ± 5.50	3.68 ± 2.96
AOFAS	Pain: 24.66 ± 4.19 Function: 31.82 ± 5.07 Alignment: 4.77 ± 1.46 Total: 61.26 ± 6.97	Pain: 36.08 ± 2.78* Function: 40.39 ± 3.18* Alignment: 12.43 ± 1.49* Total:88.90 ± 4.68*

*, compared with preoperative values, *P* < 0.01

### Surgical method

**Fig. 1:**
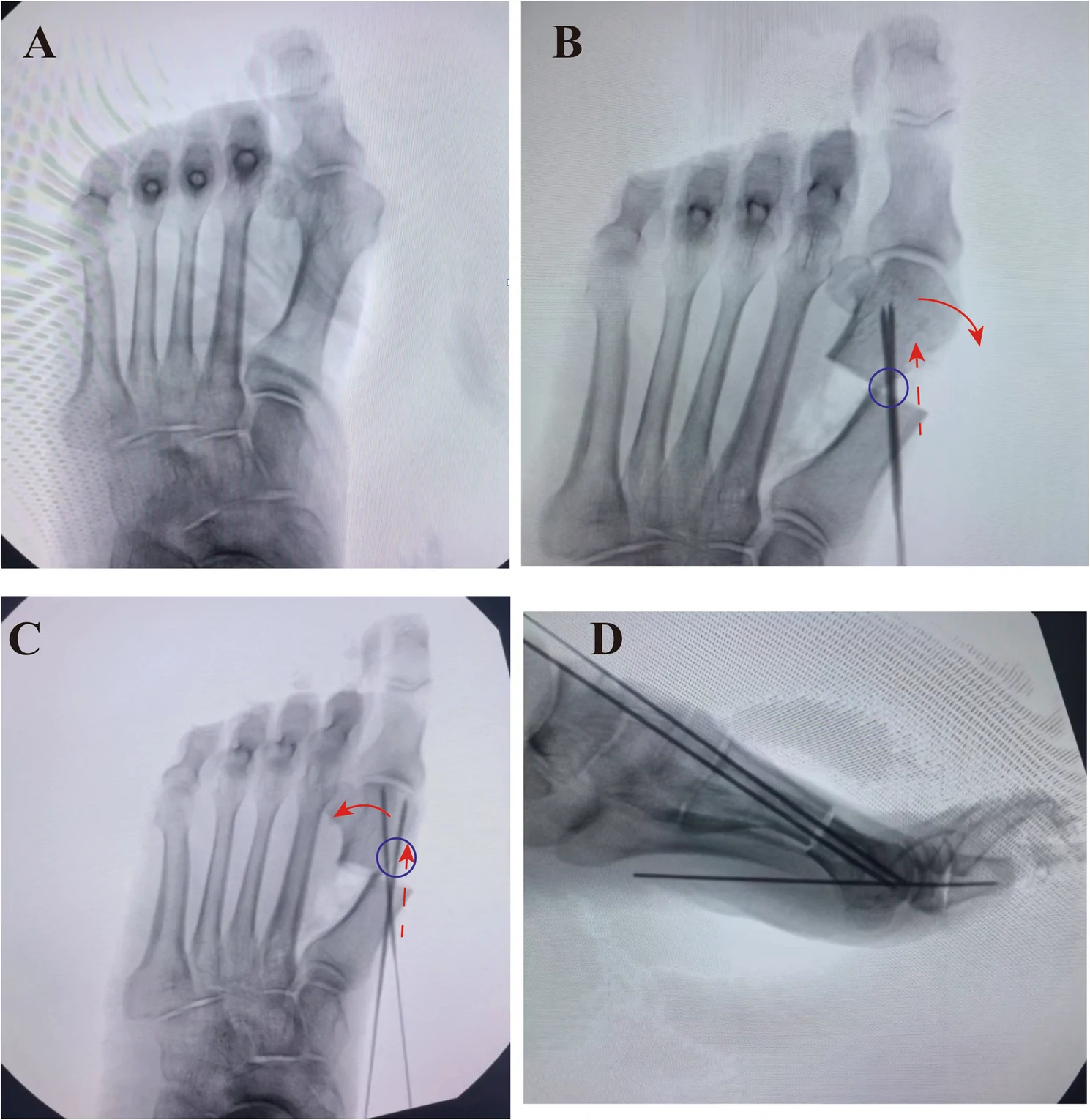
To avoid first metatarsophalangeal joint incongruence (the seesaw effect), the K-wire should be inserted as distally as possible within the distal metatarsal. Preoperative radiograph of moderate hallux valgus is shown in (**A**). When positioned too proximally (near the osteotomy line), drilling causes the metatarsal head to tilt inward, leading to joint malalignment (**B**). In contrast, distal placement allows the head to evert properly during drilling, preserving joint congruity (**C**). Lateral view shows K-wires distributed along the dorsal and plantar aspects of the metatarsal axis (**D**)

First, an approximately 1 cm incision was made at the base of the proximal phalanx. The medial joint capsule of the first MTP joint was dissected using a periosteal elevator. An oscillating saw was then used to excise the medial eminence (bunion) along the longitudinal axis of the first metatarsal. A 1.5 cm longitudinal incision was made at the proximal first metatarsal neck to expose the first metatarsal, followed by a transverse osteotomy performed perpendicular to the second metatarsal shaft using a mini oscillating saw. After osteotomy, a periosteal elevator was inserted into the proximal medullary canal to translate the fragment medially while depressing the metatarsal head to reconstruct the transverse arch. Simultaneously, the hallux manually rotated to neutral to correct pronation. Fixation was achieved with two 1.5 mm K-wires inserted via the modified K-wire fixation technique: from the medial cortex of the proximal fragment, passes through the medullary canal, reaches the medial cortex of the distal fragment, and then advances further to penetrate the lateral cortex of the distal fragment. At this point, the first MTP joint is routinely mobilized to assess joint motion. If the K-wire penetrates the lateral cortex without causing joint impingement or clicking, we retain this position—in which case the wire can safely penetrate the lateral cortex. If joint impingement or clicking occurs, we retract the K-wire appropriately until smooth joint motion is restored. Two K-wires are distributed along the dorsal and plantar aspects of the first metatarsal shaft axis. When inserting the K-wires into the distal metatarsal, it should be placed as distally as possible to avoid first metatarsophalangeal joint incongruence, known as the “seesaw effect” (Fig. 1).

**Fig. 2:**
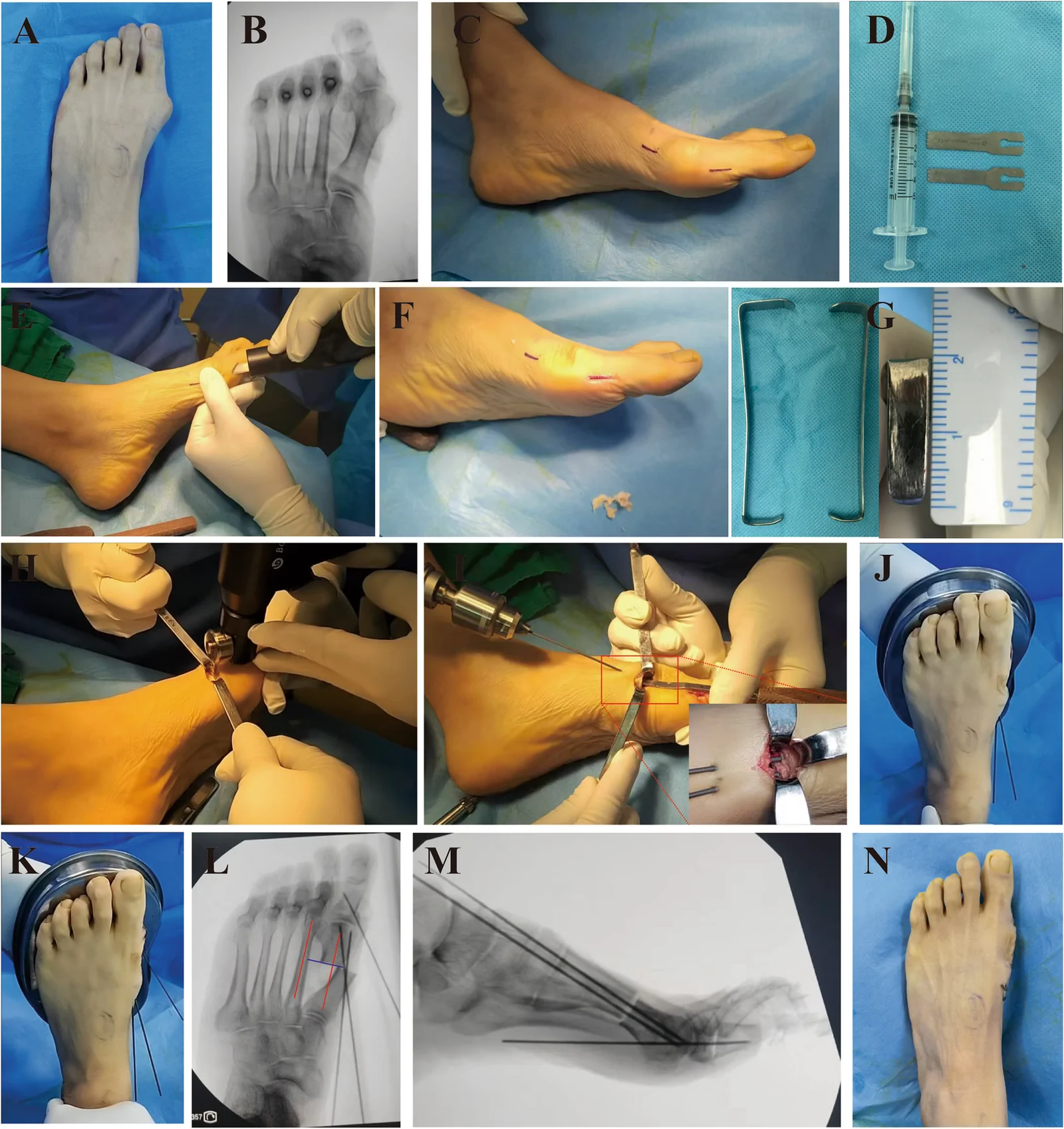
The modified fourth-generation minimally invasive technique combined with modified K-wire fixation. **A**-**B**: Preoperative clinical photograph and anteroposterior (AP) weight-bearing radiograph demonstrating hallux valgus deformity with pronation of the hallux. **C**: Preoperative skin marking of the planned surgical incision. **D**: Photograph of the mini-oscillating saw blade. **E**: The medial eminence (bunion) and osteophytes are excised along the longitudinal axis of the first metatarsal using the mini-oscillating saw. **F**: Excised joint capsule and bone fragments; note the elimination of the medial prominence. **G**: Photograph of the small retractor. **H**: A transverse osteotomy of the first metatarsal is performed using the oscillating saw, ensuring the cut is perpendicular to the axis of the second metatarsal. **I**: The first metatarsal head is translated laterally, a periosteal elevator is used to shift the proximal metatarsal fragment medially. Fixation is achieved using two K-wires, ensuring the K-wires sequentially penetrate the medial cortex of the proximal fragment and then the medial cortex of the metatarsal head. **J**: Intraoperative weight-bearing assessment reveals residual mild hallux valgus. **K**: Following Akin osteotomy, repeat intraoperative weight-bearing assessment shows resolution of the residual hallux valgus deformity. **L**: Fluoroscopic image confirming satisfactory correction of the hallux valgus deformity. The axis of the first metatarsal is parallel to the second metatarsal axis (note red line), and the osteotomy line is perpendicular to the shaft of the second metatarsal (note blue line). **M**: Lateral fluoroscopic view showing the K-wires positioned plantarly and dorsally relative to the axis of the first metatarsal. **N**: Postoperative clinical photograph after wound closure

Correction was assessed under both static and dynamic conditions (simulated weight-bearing by applying pressure to the first and fifth metatarsal heads). Dynamic assessment was performed by manually applying simultaneous pressure to the first and fifth metatarsal heads toward the plantar direction to simulate weight-bearing. The hallux position was evaluated: if a residual valgus angle > 5° or obvious deviation from neutral was observed under dynamic testing, it was defined as inadequate correction, triggering adjunctive procedures. This assessment was performed by the same surgeon, and intraoperative reproducibility was confirmed. If malalignment or residual deformity persisted, percutaneous lateral soft tissue release was performed. If residual valgus remained, an Akin osteotomy was added (Fig. 2). For those with a hammer toe deformity of the second toe, a closed wedge osteotomy of the second metatarsal and extension of the extensor tendon of the toe are performed.

The prominent proximal metatarsal was resected using a mini oscillating saw, and the excised bone was packed laterally at the osteotomy site to promote healing. A mixture was prepared by combining 1 ml of Compound Betamethasone Injection (Hangzhou MSD Pharmaceutical Co., Ltd.), 10 ml of Ropivacaine Hydrochloride Injection (Guangdong Jiabo Pharmaceutical Co., Ltd.), and 50 mg of Flurbiprofen Injection (Wuhan Yanda Life Sciences Co., Ltd.). Subsequently, an appropriate portion of the mixture was injected into the periosteum, joint capsule, and subcutaneous tissue for analgesia [11]. The incision was closed in layers, followed by compressive bandaging with a toe separator placed in the first intermetatarsal space.

### Postoperative management

Postoperatively, oral NSAIDs were administered. K-wires removed at 6 weeks. Patients walked in a forefoot-offloading shoe at 4–6 h post-surgery, transitioned to regular footwear at 8 weeks, and resumed sports at 3 months. Weight-bearing radiographs and clinical assessments were scheduled at 6 weeks, 3, 6, and 12 months.

### Observation indicators of clinical efficacy evaluation

Record the number of intraoperative fluoroscopy shots, incision healing status, and osteotomy healing status. Get weight-bearing X-rays before surgery and at 3, 6, and 12 months after surgery, measuring changes in HVA, IMA, and DMAA indicators. The clinical efficacy is evaluated using the American Orthopaedic Foot and Ankle Society MTP-InterPhalangeal scale (AOFAS MTP-IP) [12].

### Statistical analysis

Statistical analyses were performed using SPSS version 22.0 (IBM, Armonk, NY, USA). Continuous data are presented as mean±standard deviation. Normality and homogeneity of variance were assessed prior to group comparisons. Depending on data distribution, either the unpaired t-test (with or without Welch’s correction) or the Mann-Whitney rank-sum test was applied. P value < 0.05 was considered statistically significant.

## Results

### Results of finite element analysis

Analysis of Von Mises stress distribution revealed distinct patterns between the two fixation techniques. In the “in-out-in” group, stress concentrated at the metatarsal head, while in the modified K-wire fixation group, stress was primarily distributed around the osteotomy site. No significant differences in stress magnitude were observed at 4 mm and 8 mm translation, though at 12 mm, the modified technique showed higher osteotomy site stress (35.252 MPa vs. 26.355 MPa).

K-wire stress increased with greater translation distances. The modified fixation technique demonstrated significantly lower K-wire stress at both 4 mm (14.440 MPa vs. 42.406 MPa) and 12 mm (115.840 MPa vs. 193.630 MPa) translation, suggesting reduced pin fracture risk. Stress was predominantly concentrated at the osteotomy site in both groups.

**Fig. 3:**
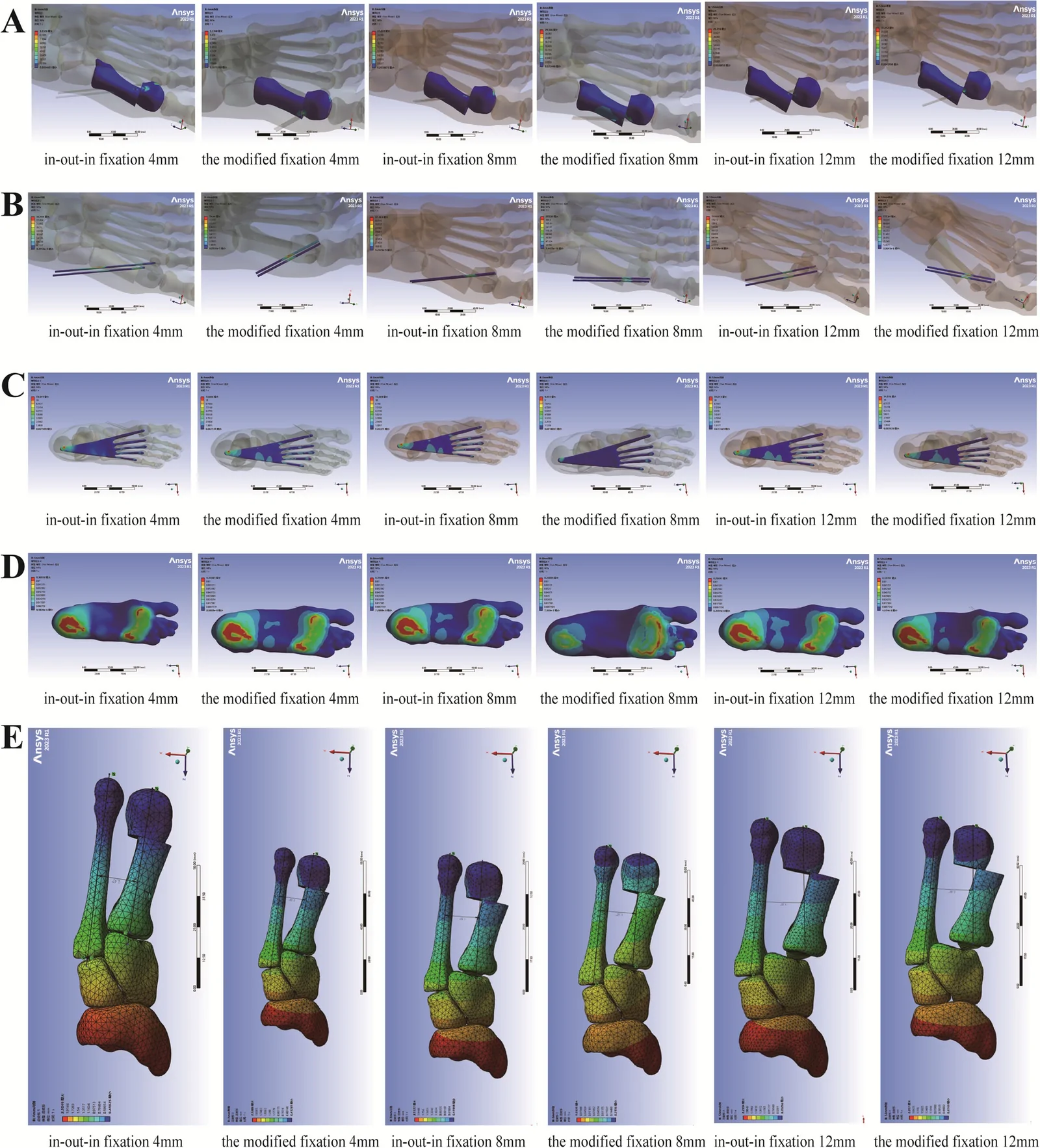
The results of FEA between “In-Out-In” versus modified K-wire fixation. **A**, stress nephograms at the osteotomy site for the two fixation techniques at 4 mm, 8 mm, and 12 mm of translation. **B**, stress nephograms on the Kirschner wires for the two fixation techniques at 4 mm, 8 mm, and 12 mm of translation. **C**, stress nephograms of the plantar fascia for the two fixation techniques at 4 mm, 8 mm, and 12 mm of translation. **D**, stress nephograms of the plantar load-bearing areas for the two fixation techniques at 4 mm, 8 mm, and 12 mm of translation. **E**, Intermetatarsal angle (IMA) measurements for the two fixation techniques at 4 mm, 8 mm, and 12 mm of translation

No significant differences were found between techniques regarding plantar fascia stress, plantar load-bearing distribution, or IMA correction. Both fixation methods restored physiological plantar loading patterns without abnormal stress concentration. Three-dimensional displacement analysis confirmed comparable initial stability between techniques, with minimal displacement (< 0.1 mm) across all translation distances. Excessive translation resulted in negative IMA values, indicating potential hallux varus risk. Detailed data are presented in Table 2; Fig. 3.

**Table 2:** Finite element analysis of two fixation techniques under different translation distances

	In-Out-In fixation			The modified K-wire fixation		
	4 mm	8 mm	12 mm	4 mm	8 mm	12 mm
Von Mises stress of first metatarsal (MPa)	9.133	27.222	26.355	9.537	29.566	35.252
Von Mises stress of K-wire (MPa)	42.406	97.363	193.630	14.440	102.960	115.840
Plantar Fascia Stress (MPa)	13.691	13.683	14.043	13.666	13.955	14.176
Plantar Load-Bearing Area Stress (MPa)						
Heel	0.101	0.110	0.095	0.110	0.103	0.098
1st MT head	0.073	0.084	0.083	0.076	0.087	0.074
5st MT head	0.091	0.092	0.084	0.093	0.090	0.081
Displacement of Distal Metatarsal Head						
X-axis (mm)	0.001	-0.013	-0.048	0.010	0.128	0.032
Y-axis (mm)	0.617	0.628	0.591	0.635	0.819	0.614
Z-axis (mm)	-0.264	-0.258	-0.167	-0.263	-0.140	-0.105
IMA (°)	2.432	-1.79	-5.953	2.288	-1.574	-5.681

### Results of clinical efficacy evaluation

A total of 108 patients (164 feet) with a mean follow-up of 18.90 ± 3.57 (13–25) months were enrolled, including 38 mild, 70 moderate, and 56 severe HV cases. All incisions healed primarily. Two patients developed superficial pin site infections, which resolved with outpatient care, and two reported transient metatarsalgia that improved with arch support. No cases of K-wire breakage or migration, malunion, hallux varus, or deep infection were observed. During K-wire removal at 6 weeks, no wire breakage or retained fragments occurred. Complete bony union was achieved in all patients at one year postoperatively.

Significant postoperative improvements were observed across all radiographic and functional outcomes (all *P* < 0.01). Overall, HVA improved from 36.93°±9.32° to 9.57°±4.88°, IMA from 14.86°±3.82° to 6.08°±3.43°, and AOFAS score from 61.26 ± 6.97 to 88.90 ± 4.68. Subgroup analysis by severity demonstrated consistent correction across all groups. In severe HV patients, HVA improved from 47.33°±5.85° to 12.59°±4.92°, IMA from 17.32°±3.52° to 5.80°±3.60°, and AOFAS score from 62.54 ± 6.52 to 89.48 ± 4.10 (Table 3).

**Table 3:** Preoperative and postoperative outcomes of patients with different grades of HV

	Pre-operation	post-operation
mild HV	HVA: 25.48 ± 3.22 IMA: 12.12 ± 3.00 DMAA: 8.82 ± 4.62 AOFAS Pain: 23.66 ± 4.82 Function: 30.32 ± 4.85 Alignment: 4.97 ± 1.33 Total: 58.95 ± 7.22	HVA: 6.12 ± 2.34* IMA: 6.46 ± 3.67* DMAA: 3.23 ± 2.73* AOFAS Pain: 36.05 ± 2.49* Function: 40.32 ± 3.48* Alignment: 12.53 ± 1.46* Total: 88.89 ± 4.14*
moderate HV	HVA: 34.82 ± 2.88 IMA: 14.38 ± 3.21 DMAA: 10.81 ± 5.18 AOFAS Pain: 24.29 ± 4.28 Function: 32.53 ± 4.98 Alignment: 4.67 ± 1.49 Total: 61.49 ± 6.58	HVA: 9.03 ± 4.40* IMA: 6.09 ± 3.12* DMAA: 3.79 ± 3.23* AOFAS Pain: 35.70 ± 3.22* Function: 40.50 ± 3.21* Alignment: 12.23 ± 1.54* Total: 88.43 ± 5.30*
severe HV	HVA: 47.33 ± 5.85 IMA: 17.32 ± 3.52 DMAA: 12.20 ± 6.07 AOFAS Pain: 25.82 ± 3.24 Function: 31.95 ± 5.11 Alignment: 4.77 ± 1.50 Total: 62.54 ± 6.52	HVA: 12.59 ± 4.92* IMA: 5.80 ± 3.60* DMAA: 3.85 ± 2.70* AOFAS Pain: 36.57 ± 2.23* Function: 40.30 ± 2.93* Alignment: 12.61 ± 1.41* Total: 89.48 ± 4.10*

*, compared with preoperative values, *P* < 0.01

**Fig. 4:**
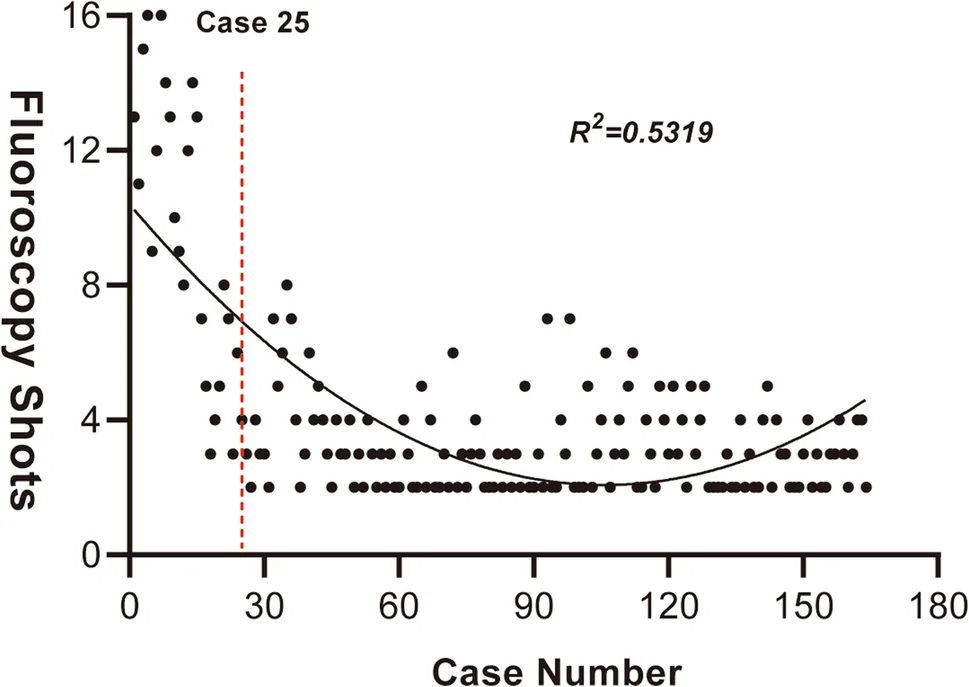
Learning curve for intraoperative fluoroscopy usage in the modified fourth-generation minimally invasive technique. Derivative of the equation solved for overall slope of the data set identified the learning curve at case 25

**Fig. 5:**
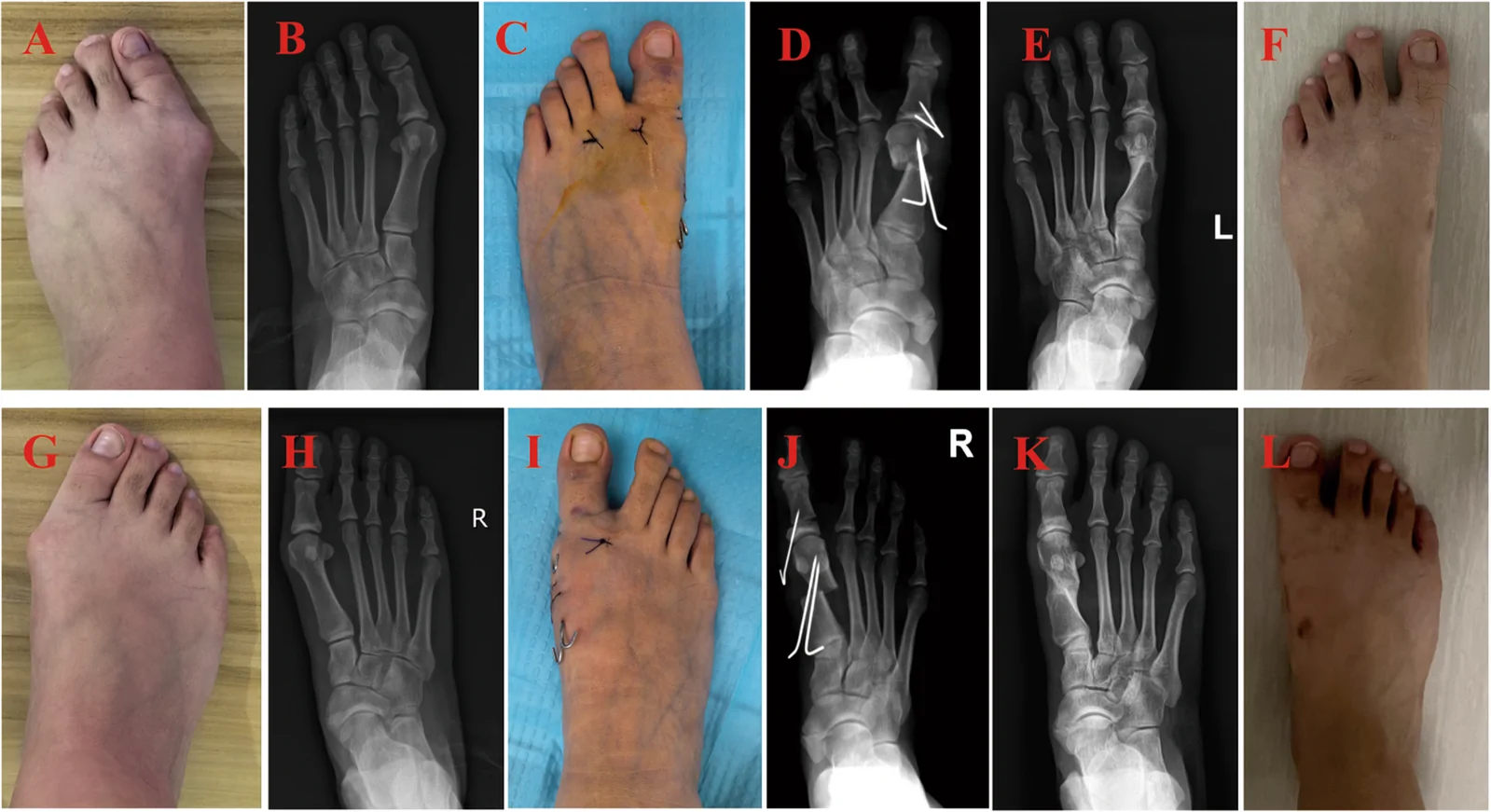
Case 1, **A**: Preoperative clinical photograph of the left foot; **B**: Preoperative anteroposterior weight-bearing radiograph of the left foot. **C**: Postoperative clinical photograph of the left foot. **D**: Postoperative day 2 follow-up AP radiograph of the left foot. **E**: 1-year postoperative follow-up AP weight-bearing radiograph of the left foot. **F**: Postoperative clinical photograph of the left foot. **G**: Preoperative clinical photograph of the right foot. **H**: Preoperative AP weight-bearing radiograph of the right foot. **I**: Postoperative clinical photograph of the right foot. **J**: Postoperative day 2 follow-up AP radiograph of the right foot. **K**:1-year postoperative follow-up AP weight-bearing radiograph of the right foot. **L**: 1-year postoperative clinical photograph of the right foot

**Fig. 6:**
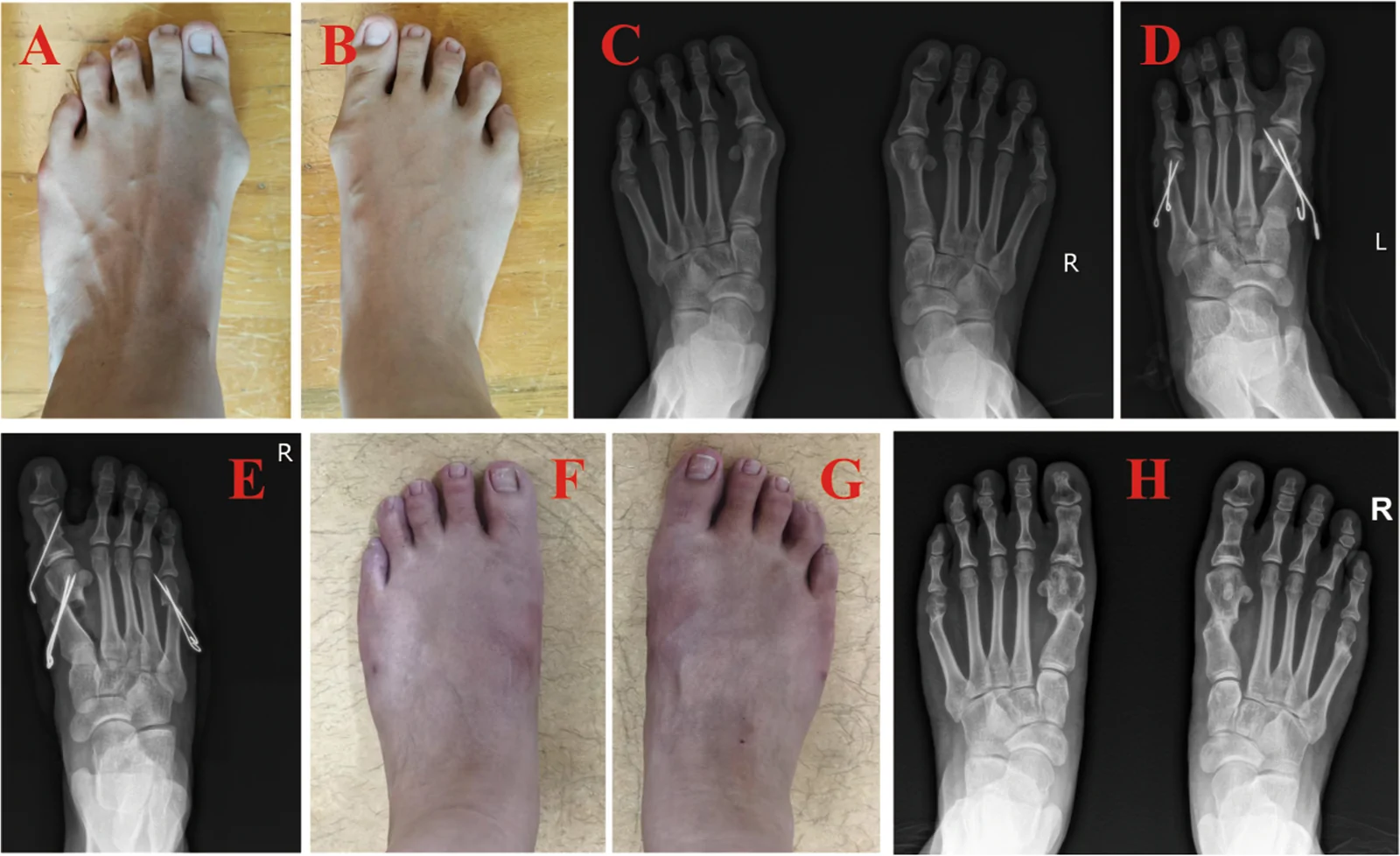
Case 2, **A**: Preoperative clinical photograph of the left foot. **B**: Preoperative clinical photograph of the right foot. **C**: Preoperative weight-bearing AP radiograph of both feet. **D**: Postoperative day 2 follow-up AP radiograph of the left foot. **E**: Postoperative day 2 follow-up AP radiograph of the right foot. **F**: 1-year postoperative clinical photograph of the left foot. **G**: 1-year postoperative clinical photograph of the right foot. **H**: 1-year postoperative weight-bearing AP radiograph of both feet

**Fig. 7:**
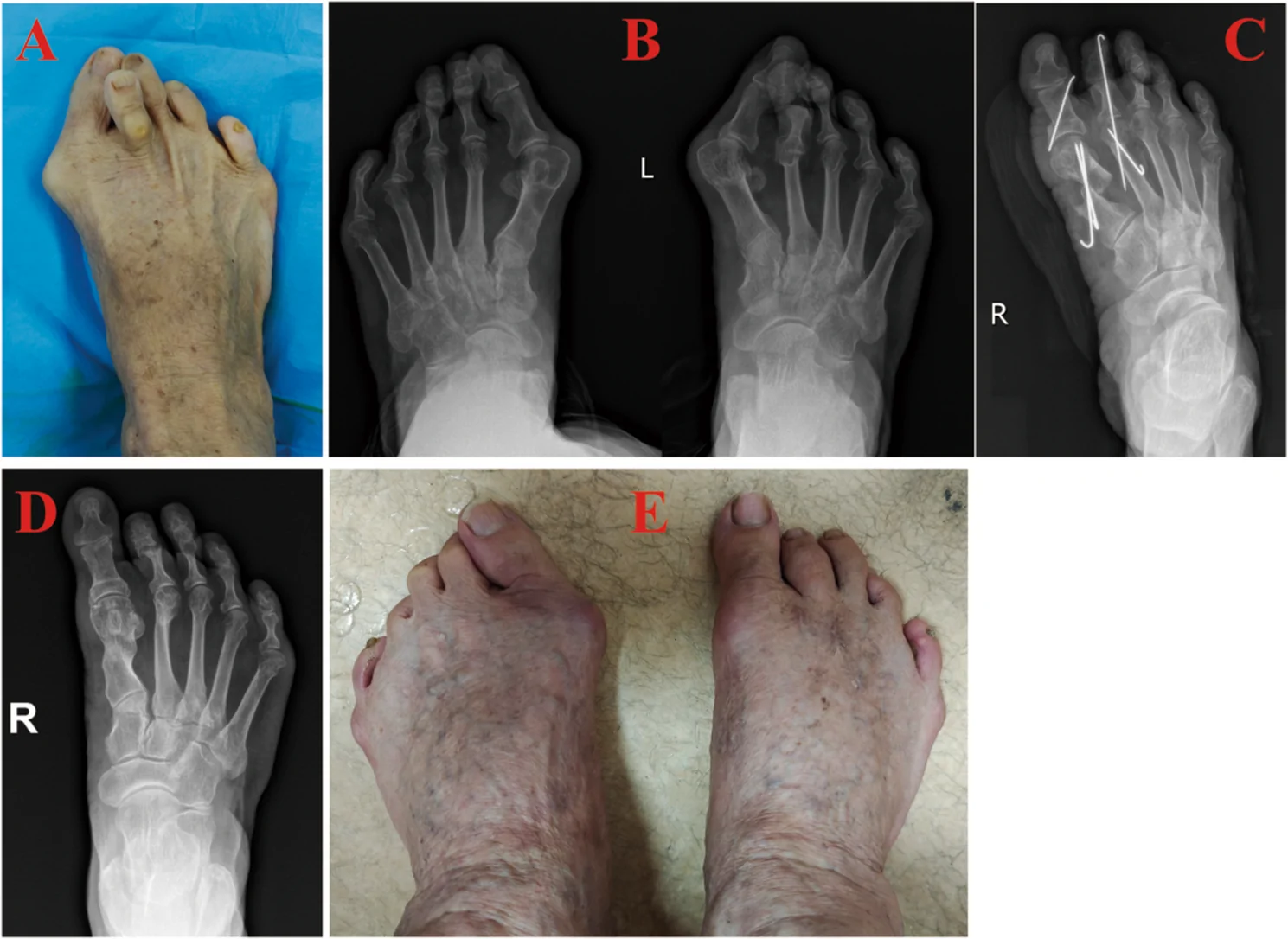
Case 3, **A**: Preoperative clinical photograph of the right foot. **B**: Preoperative weight-bearing AP radiograph of both feet. **C**: Postoperative day 2 follow-up AP radiograph of the right foot. **D**: 1-year postoperative weight-bearing AP radiograph of right feet. **E**: 1-year postoperative clinical photograph of the both feet

Mean intraoperative fluoroscopy was 4.14 ± 3.04 (range, 2–16) shots per foot. A learning curve was observed: the first 24 cases averaged 9.71 ± 4.04 shots, decreasing to 3.19 ± 1.35 shots for the subsequent 140 procedures after stabilization at approximately 25 cases. Over 50% of procedures required ≤ 3 shots, and > 30% required only 2 shots for K-wire verification. The learning curve is illustrated in Figs. 4, and representative cases of HV correction at different severity grades are shown in Fig 5, 6, and 7.

## Discussion

With the introduction of third and fourth-generation techniques, their clinical application has become increasingly widespread in the treatment of HV. Although numerous studies have explored the biomechanical properties of different treatments for HV, comparative research on different K-wire fixation techniques remains relatively scarce [13–16].

Based on the fourth-generation technique, we developed a modified K-wire fixation method, designated as the modified K-wire fixation technique. Compared to the “in-out-in” technique, the K-wire can be seen passing through the proximal metatarsal head in the modified technique, and the first MTP joint can be assessed to determine whether the K-wire has crossed the MTP joint, allowing the entire implantation process to happen with less fluoroscopy. However, the biomechanical differences between these two K-wire fixation techniques were previously unclear.

Our results revealed that at 4 mm and 8 mm of translation, the Von Mises stress on the first metatarsal showed no significant difference between the modified K-wire fixation and “in-out-in” techniques. However, the Von Mises stress on the K-wires themselves was significantly lower in the modified group, suggesting a reduced risk of pin breakage compared to the “in-out-in” technique. Regarding plantar fascia stress, no significant differences were observed between the two groups at any translation distance (4, 8, 12 mm). The displacement data at the osteotomy site further indicated no significant differences between the two fixation techniques across the different translation distances, implying that their fixation strengths are comparable. The comparable stability between the modified K-wire fixation and “in-out-in” techniques can be explained by their similar biomechanical principle. The modified K-wire fixation creates a three-point support construct: two points on the medial cortex (proximal and distal fragments) and one point on the lateral cortex of the distal fragment. This configuration resists shear and rotational forces effectively, analogous to the three-cortical fixation of the “in-out-in” technique, which engages the medial and lateral cortices of the proximal fragment and the lateral cortex of the distal fragment. Thus, the reduced pin stress in the modified technique does not compromise stability.

K-wire fixation is increasingly used in HV correction. Traditionally, K-wires were employed in the Bösch technique [17], where fixation was achieved through the intramedullary canal of the proximal metatarsal. This method fell out of favor due to insufficient fixation strength, poor rotational control, and high complication rates [18]. Subsequently, some scholars applied K-wire fixation in modified Chevron osteotomies, with biomechanical studies suggesting that three-cortical K-wire fixation provides stability comparable to single-screw fixation [19]. Mohammed et al. demonstrated that K-wire fixation serves as a viable strategy in proximal metatarsal osteotomy. This method offered clinical outcomes equivalent to those of plate and screw constructs but was more cost-effective and associated with a lower rate of reoperation [20]. With the clinical adoption of third and fourth-generation transverse metatarsal osteotomies, K-wires have regained popularity for stabilizing the osteotomy site. Kim et al. reported that augmenting single-screw fixation with a K-wire can better correct the DMAA and reduce the recurrence of HV [21]. However, to the best of our knowledge, clinical studies on minimally invasive osteotomy correction utilizing K-wire fixation alone have not yet been reported.

All osteotomy sites healed uneventfully, with K-wires removed at 6 weeks and bony union was achieved at the osteotomy site in all patients at 1 year. A key technical consideration is distal K-wire placement within the metatarsal head: directing the wire away from the osteotomy site prevents the “seesaw effect” (intraoperative head inversion and subsequent MTP joint malalignment), whereas proximal placement predisposes to this complication.

In this study, the mean number of fluoroscopy shots per foot for HV correction was 4.14. During the first 24 correction procedures (cases 1–24), the mean fluoroscopy shots was 9.71 times. As surgical experience increased, intraoperative fluoroscopy usage gradually decreased, with a mean of 3.19 shots for procedures 25–164. The inflection point of the learning curve occurred at approximately 25 cases, which is comparable to the learning curve observed for the MICA technique, where the inflection point has been reported around the 29th–30th case [8]. Notably, the fluoroscopy frequency required for the modified fourth-generation minimally invasive technique was significantly lower than that of the “in-out-in” fixation technique, which reported requires over 60 shots [22]. This suggests that the modified fourth-generation minimally invasive technique can effectively reduce intraoperative fluoroscopy exposure. However, it is important to note that this comparison is indirect, as we did not perform a contemporaneous head-to-head trial with the “in-out-in” or MICA techniques. Direct comparative studies are warranted to confirm the superiority of our method regarding fluoroscopy reduction.

The direction of the osteotomy determines whether there will be shortening of the hallux postoperatively. Both the third and fourth-generation minimally invasive techniques suggest doing the osteotomy vertically on the first metatarsal shaft, slightly favoring the distal side, which helps make up for the 2 mm bone loss from the Shannon drill during the osteotomy and preventing shortening of the hallux [23]. In the modified fourth-generation minimally invasive surgery, since we use a miniature oscillating saw for the osteotomy, it has almost no impact on the length of the first metatarsal. Performing the osteotomy perpendicular to the second metatarsal shaft allows for better parallel alignment of the first and second metatarsals after shifting, improving the IMA angle.

How to prevent recurrence of HV deformity after correction surgery is a key concern for foot and ankle surgeons during the procedure. We propose a new dimension to assess whether the correction of HV deformity has been adequately achieved. In addition to correcting the coronal plane HV, sagittal plane elevation of the first metatarsal head, and transverse plane pronation deformity of the hallux, it is also necessary to consider whether the HV deformity has improved in both dynamic and static aspects, referred to as the fourth dimension of correction. The static aspect refers to assessing whether there is any residual HV deformity after the internal fixation is implanted; the dynamic assessment refers to evaluating whether the HV deformity maintains a neutral position while simulating weight-bearing. If the hallux still shows valgus in the dynamic aspect, the risk of postoperative recurrence goes up, necessitating adequate soft tissue release or additional procedures, such as lateral adductor release, additional Akin surgery, or insufficient metatarsal head shifting. If the hallux shows varus in the dynamic aspect, it may indicate excessive shifting of the metatarsal head or excessive soft tissue release laterally.

This study has several key advantages. First, FEA confirmed that the modified K-wire fixation technique provides comparable stability to conventional “in-out-in” fixation while significantly reducing pin fracture risk, offering a strong biomechanical foundation for clinical application. Second, unlike third- and fourth-generation methods requiring specialized drills, our modified technique utilizes a standard mini-oscillating saw or K-wire drilling, enhancing accessibility in resource-limited settings. Third, direct visualization of K-wire passage through the medial cortex minimizes fluoroscopy dependence, reducing radiation exposure and operative time. Fourth, K-wire fixation eliminates the need for costly implants or secondary removal surgery, lowering healthcare costs and improving patient acceptance.

Despite these strengths, several limitations should be acknowledged. The finite element model was derived from a single patient’s CT data, limiting generalizability, and simplified soft tissue modeling may not fully replicate in vivo biomechanics. Nevertheless, the selected patient had moderate HV (HVA 37°, IMA 15°) and represented a typical case, with first metatarsal morphology consistent with most clinical presentations. Moreover, the primary purpose of our FEA was to compare the relative biomechanical differences between the two K-wire fixation techniques rather than to predict absolute stress values; therefore, a single-patient model still provides valid comparative insight. Additionally, FEA alone cannot replace experimental validation. No cadaveric or laboratory biomechanical studies were conducted to corroborate the FEA findings, which represents a limitation of this study.

In addition, this study is a retrospective case series (Level IV evidence) without a control group. All patients were treated by a single surgeon using the same technique during the study period, and no contemporaneous comparison with alternative procedures was performed. Therefore, selection bias cannot be fully excluded, and the reported outcomes should be interpreted with caution. Future prospective randomized controlled trials are needed to further validate the efficacy of this modified technique. Furthermore, the AOFAS score, while widely used, is a clinician-administered instrument and has well-documented limitations, including a potential ceiling effect and lack of patient-reported outcome measures (PROMs) such as the Manchester-Oxford Foot Questionnaire (MOXFQ) or the Foot and Ankle Outcome Score (FAOS). The absence of validated PROMs in this study is an important limitation.

## Conclusion

In summary, this study demonstrates that the modified fourth-generation minimally invasive technique, which incorporates an extra-articular transverse osteotomy, the modified K-wire fixation technique, and the principle of fourth-dimensional correction, is a safe and effective approach for managing HV across a spectrum of deformity severities.

## Supplementary Information

Supplementary Material 1.

## Data Availability

All data generated or analyzed during this study are included in this published article.

## References

[1] Holme TJ, et al. Third-Generation Minimally Invasive Chevron Akin Osteotomy for Hallux Valgus. Foot Ankle Int. 2020;41(1):50–6. 10.1177/1071100719874360 31522534

[2] Del Vecchio JJ, Ghioldi ME. Evolution of Minimally Invasive Surgery in Hallux Valgus. Foot Ankle Clin. 2020;25(1):79–95. 10.1016/j.fcl.2019.10.010 31997749

[3] Trnka HJ. Percutaneous, MIS and open hallux valgus surgery. EFORT Open Rev. 2021;6(6):432–8. 10.1302/2058-5241.6.210029 PMC8246107 34267933

[4] Lai MC, et al. Clinical and Radiological Outcomes Comparing Percutaneous Chevron-Akin Osteotomies vs Open Scarf-Akin Osteotomies for Hallux Valgus. Foot Ankle Int. 2018;39(3):311–7. 10.1177/1071100717745282 29241361

[5] Li G, et al. Clinical guideline on the third generation minimally invasive surgery for hallux valgus. J Orthop Translat. 2024;45:48–55. 10.1016/j.jot.2023.10.009 PMC10945049 38500804

[6] Lewis TL, et al. Current concepts of fourth-generation minimally invasive and open hallux valgus surgery. Bone Joint J. 2025;107–B(1):10–8. 10.1302/0301-620X.107B1.BJJ-2024-0597.R2 39740690

[7] Lewis TL, et al. Fourth-Generation Minimally Invasive Hallux Valgus Surgery With Metaphyseal Extra-Articular Transverse and Akin Osteotomy (META): 12 Month Clinical and Radiologic Results. Foot Ankle Int. 2023;44(3):178–91. 10.1177/10711007231152491 36788732

[8] Merc M, Fokter SK, I IS. Learning curve in relation to radiation exposure, procedure duration and complications rate for Minimally Invasive Chevron Akin (MICA) osteotomy. BMC Musculoskelet Disord. 2023;24(1):575. 10.1186/s12891-023-06706-1 PMC10350254 37454046

[9] Biz C et al. The Learning Curve of Reverdin-Isham and Akin Percutaneous Osteotomies for Hallux Valgus Correction: A Bayesian Approach. J Clin Med, 2025;14(6):1921–1934. 10.3390/jcm14061921 PMC11942651 40142729

[10] Cheung JT, Zhang M. A 3-dimensional finite element model of the human foot and ankle for insole design. Arch Phys Med Rehabil. 2005;86(2):353–8. 10.1016/j.apmr.2004.03.031 15706568

[11] Xie J et al. Pain Management of Hallux Valgus Surgery Is Achieved by Cocktail Therapy. Evid Based Complement Alternat Med. 2022;2022:1084815. 10.1155/2022/1084815 PMC9463003 36091582

[12] Kitaoka HB, et al. Clinical rating systems for the ankle-hindfoot, midfoot, hallux, and lesser toes. Foot Ankle Int. 1994;15(7):349–53. 10.1177/107110079401500701 7951968

[13] Lewis TL, et al. Comparative biomechanical study of different screw fixation methods for minimally invasive hallux valgus surgery: A finite element analysis. Foot Ankle Surg. 2025;31(2):160–9. 10.1016/j.fas.2024.09.001 39261184

[14] Xie Q, Li X, Wang P. Three dimensional finite element analysis of biomechanics of osteotomy ends with three different fixation methods after hallux valgus minimally invasive osteotomy. J Orthop Surg (Hong Kong). 2023;31(2):10225536231175235. 10.1177/10225536231175235 37167369

[15] Mao R, et al. Biomechanical study on surgical fixation methods for minimally invasive treatment of hallux valgus. Med Eng Phys. 2017;46:21–6. 10.1016/j.medengphy.2017.04.010 28527835

[16] Li Y, et al. Biomechanical Comparison between Rotational Scarf Osteotomy and Translational Scarf Osteotomy: A Finite Element Analysis. Orthop Surg. 2023;15(12):3243–53. 10.1111/os.13903 PMC10694014 37731316

[17] Bernasconi A, et al. Bosch osteotomy for hallux valgus correction: results at a mean 10-year follow-up. Arch Orthop Trauma Surg. 2023;143(3):1293–300. 10.1007/s00402-021-04259-3 34839385

[18] Consensus on Surgical Management of Hallux Valgus from China. Orthop Surg. 2015;7(4):291–6. 10.1111/os.12207 PMC6584274 26819077

[19] Chen J, et al. Biomechanical Comparison of a Novel Method of Tricortical Kirschner Wire Fixation of Distal Chevron Osteotomies of the First Metatarsal Versus Traditional Kirschner Wire and Versus Screw Fixation. Foot Ankle Spec. 2022;15(5):426–31. 10.1177/1938640020965339 33090018

[20] Mohammed A et al. Evaluation of K-wires splinting of first and second metatarsals following proximal open wedge osteotomy for hallux valgus correction. J Foot Ankle Surg. 2025;S1067-2516(25):00276–5. 10.1053/j.jfas.2025.09.009 41016619

[21] Kim J, et al. Radiographic Comparison of Open and Minimally Invasive Distal Chevron Metatarsal Osteotomy in Patients With Hallux Valgus. J foot ankle Surg. 2024;63(3):386–91. 10.1053/j.jfas.2024.01.012 38281555

[22] Espinosa-Uribe AG, et al. A comparison of three techniques for the osteosynthesis after minimal invasive osteotomies for hallux valgus. Int Orthop. 2024;48(8):2137–43. 10.1007/s00264-024-06178-5 38589709

[23] Campillo-Recio D, et al. Fourth-Generation Minimally Invasive Hallux Valgus Surgery With Guided Percutaneous System: Step-by-Step Surgical Technique. Arthrosc Tech. 2025;14(6):103504. 10.1016/j.eats.2025.103504 PMC12255461 40656733

