# Supplementary material for: Modified fourth-generation minimally invasive technique with a modified K-wire fixation technique for hallux valgus: a biomechanical and clinical study
Source: BMC Musculoskelet Disord. 2026 Jul 28;27:908. doi: 10.1186/s12891-026-10151-1 (PMC13700298; doi:10.1186/s12891-026-10151-1)
Supplement: Supplementary file 1 [file 12891_2026_10151_MOESM1_ESM.docx]

**Supplementary Material**

**Methods:**

***2.1 Development of the HV Finite Element Model***

A 36-year-old female patient (weight: 50 kg, height: 158 cm) with moderate HV [HVA: 37°; intermetatarsal angle (IMA): 15°] was recruited. Non-weightbearing CT data were collected from this patient for the development of the finite element (FE) model. The modeling process was initiated in Mimics software (Materialise, Belgium) to extract bone information from the CT scans and create a preliminary three-dimensional (3D) model of the foot. This model was subsequently imported into Geomagic software (Geomagic Inc.) for smoothing and denoising, resulting in a solid model. The processed model was then transferred to SolidWorks (Dassault Systèmes) for the assembly of internal fixation components. Following this, it was imported into the Ansys SC module to inspect for potential issues and identify surfaces for constraint application, ultimately establishing a comprehensive FE model. Finally, the model was imported into the Ansys finite element analysis software (Ansys Inc., USA) where material properties and boundary conditions were defined to obtain the final HV FE model. Informed consent was obtained from the patient after a thorough explanation of the procedure and associated risks. The study protocol was reviewed and approved by the Ethics Committee of *** in accordance with the Declaration of Helsinki (Ethical Approval Number: ZE2025-311).

Established material properties from prior literature were assigned to cortical bone, cartilage, ligaments, the plantar fascia, and K-wires. A vertical load of 250 N was applied to the midfoot to simulate body weight during standing. Under these boundary conditions, the following outcome measures were calculated and compared between the two techniques at each translation distance: 1) stress distribution at the osteotomy site; 2) stress distribution on the K-wires; 3) three-dimensional displacement (X-, Y-, Z-axes) of the osteotomy site; 4) change in the intermetatarsal angle (IMA); and 5) stress distribution within the plantar fascia and across the plantar foot regions.

***2.2 Material Property Assignment***

Material parameters for cortical bone, cartilage, ligaments, plantar fascia, the ground support, and Kirschner wires were defined based on established parameters from previous finite element analysis studies on HV. Cortical bone and K-wires were assigned elastic moduli of 73,000 MPa and 187,500 MPa, with Poisson's ratios of 0.3 and 0.33, respectively. Articular cartilage, soft tissue, plantar fascia, and the ground support were assigned elastic moduli of 10 MPa, 1.15 MPa, 350 MPa, and 17,000 MPa, with Poisson's ratios of 0.4, 0.49, 0.4, and 0.1, respectively.

***2.3 Establishment of the Minimally Invasive Osteotomy Model***

A transverse osteotomy was performed 2 cm proximal to the first metatarsal head on the medial side, with the osteotomy plane perpendicular to the shaft of the second metatarsal. Following the osteotomy, the distal metatarsal head was laterally translated to varying degrees (4 mm, 8 mm, and 12 mm). Fixation using Kirschner wires was then applied at each of these translation distances. Group A was fixed using the "in-out-in" technique, while Group B was fixed using the modified fixation technique.

***2.4 Meshing***

Volume meshing was performed using Hypermesh software. Bones and cartilage were meshed with C3D4 tetrahedral elements. Ligaments and the plantar fascia were modeled using truss elements, and muscles were represented using connector elements capable of simulating muscle forces. For the "in-out-in" fixation group, element and node counts were 311,869 and 186,532 at 4 mm translation, 311,472 and 186,356 at 8 mm translation, and 322,897 and 193,570 at 12 mm translation, respectively. For the modified fixation group, element and node counts were 310,442 and 185,756 at 4 mm translation, 306,788 and 182,656 at 8 mm translation, and 308,537 and 184,812 at 12 mm translation, respectively. All models demonstrated consistent mesh density across the different simulation conditions.

***2.5 Boundary Conditions and Loading***

The foot model was fixed at its interface with the ground. A vertical force of 250 N was applied to the midfoot region to simulate body weight. This load was determined based on the methodology described by Cheung *et al.*^14^, who reported a ground reaction force of approximately 350 N per foot for a 70 kg individual during standing. Accordingly, a proportional load of 250 N was applied for our patient weighing 50 kg.

***2.6 Outcome Measures of Finite Element Analysis***

A spatial coordinate system was established, defined by three mutually perpendicular axes (X, Y, Z). The X-axis represents the horizontal plane, the Y-axis the vertical plane, and the Z-axis the coronal plane, parallel to the plantar plane. Under the specified constraints and loading conditions, the following parameters were calculated and compared between the "in-out-in" and modified fixation techniques at 4 mm, 8 mm, and 12 mm of lateral translation: 1) Stress distribution at the osteotomy site; 2) Stress distribution on the K-wires; 3) Displacement of the osteotomy site along the X, Y, and Z axes; 4) Change in the IMA; 5)Stress distribution within the plantar fascia and across the plantar foot regions.

**References:**

[1].	Cheung, J.T. and M. Zhang, A 3-dimensional finite element model of the human foot and ankle for insole design. Arch Phys Med Rehabil, 2005. 86(2): p. 353-8.
